# ﻿*Oreocharisguangwushanensis*, a new species of Gesneriaceae from Sichuan Province, China

**DOI:** 10.3897/phytokeys.201.77574

**Published:** 2022-06-28

**Authors:** Zheng-Long Li, Hai-Jun Ma, Zheng-Rong Ye, De-Chang Meng, Fang Wen, Xin Hong

**Affiliations:** 1 Anhui Provincial Engineering Laboratory of Wetland Ecosystem Protection and Restoration, School of Resources and Environmental Engineering, Anhui University, CN-230601, Hefei City, Anhui Province, China Anhui University Hefei China; 2 School of Ecology and Environment, Anhui Normal University, CN-241000, Wuhu city, Anhui Province, China Kunming Inst. of Botany, Chinese Academy of Sciences Kunming China; 3 The School of Life Science, Shandong Agricultural University, CN-271018, Taian City, Shandong Province, China Anhui Normal University Wuhu China; 4 Guangxi Key Laboratory of Plant Conservation and Restoration Ecology in Karst Terrain, Guangxi Institute of Botany, Guangxi Zhuang Autonomous Region and Chinese Academy of Sciences, CN-541006, Guilin City, Guangxi Zhuang Autonomous Region, China Shandong Agricultural University Taian China; 5 Gesneriad Committee of China Wild Plant Conservation Association, National Gesneriaceae Germplasm Resources Bank of GXIB, The Gesneriad Conservation Center of China, Guilin Botanical Garden, Chinese Academy of Sciences, CN-541006 Guilin, Guangxi, China Guilin Botanical Garden, Chinese Academy of Sciences Guilin China; 6 Yunnan Key Laboratory for Integrative Conservation of Plant Species with Extremely Small Populations, Kunming Inst. of Botany, Chinese Academy of Sciences, CN-650201 Kunming, Yunnan, China Guangxi Institute of Botany, Guangxi Zhuang Autonomous Region and Chinese Academy of Sciences Guilin China

**Keywords:** Didymocarpoideae, Lithophilous, new taxon, pink flowers, Sichuan flora

## Abstract

A new species of *Oreocharis*, *O.guangwushanensis* from the Sichuan Province of south-western China, is described and illustrated here. This new species has a pink corolla that is different from other species of *Oreocharis* in southwest China and, although it is morphologically similar to *O.ronganensis* and *O.reticuliflora*, it has significant differences in the colour and shape of the corolla, the apex of the corolla limb, shape and indumentum of the filaments and a shorter pistil. A detailed description, colour photographs, distribution and habitat, as well as the IUCN conservation status, are also provided.

## ﻿Introduction

After *Oreocharis* Bentham was redefined by Möller et al. (2011), it has become a large and morphologically diverse genus in Gesneriaceae Rich. & Juss. (Möller et al. 2011, 2014; Chen et al. 2014). There are 136 species and 15 varieties of *Oreocharis* in China, mainly in southern and south-western China (Wen et al. 2021; Yang and Shi 2021; GRC. 2022). Other species are distributed in north-eastern India, northern Vietnam, Japan, Myanmar, Bhutan and Thailand (Wang et al. 1990, 1998; Li and Wang 2004; Möller et al. 2017, 2018; Xu et al. 2017; Chen et al. 2018; Wei 2018).

In July 2020, three authors (ZLL, HJM, ZRY), found this plant in Guangwushan Provincial Nature Reserve during their plant diversity survey. They found this species growing on a large boulder by the roadside, but with only a small number of individuals. On the same day, they found plants of this species in flower at the bottom of a cliff. This population had more individuals than the population on the boulder, with many individuals in flower and fruit, from which they collected specimens. After further investigation, they found that there were only two populations in the Guangwushan Reserve. The author (HX) has seen this plant before, but mistakenly thought it was *Oreocharisfarreri* (Craib) Mich.Möller & A.Weber, based on its vegetative habit because it was not in flower.

After consulting some Gesneriaceae monographs (Li and Wang 2004; Wei et al. 2010) and comparing the species with other described congeners (Wang et al. 1990, 1998; Chen et al. 2016; Wei et al. 2016; Do et al. 2017; Li et al. 2017; Yang et al. 2017; Guo et al. 2018; Möller et al. 2018; Pan et al. 2019; Yang et al. 2019; Cai and Dao 2020; Chen et al. 2020; Qin et al. 2020) and specimens of Gesneriaceae deposited at IBSC, IBK, KUN, PE, US and VMN, we referenced the older specimen information and articles from other species. Then we confirmed that it is a new species.

## ﻿Materials and methods

The measurements and morphological characteristics of the new species were taken from the type specimens processed by the authors. We examined *Oreocharis* specimens in IBSC, IBK, KUN, PE, US and VMN, to find species that are morphologically similar. We confirmed it to be an undiscovered species. The type specimens of the new species were deposited in IBK and AHU and living individuals were cultivated at the Gesneriad Conservation Center of China. All morphological characters were studied under a dissecting microscope and were described using the terminology used by Wang et al. (1998).

## ﻿Taxonomic treatment

### ﻿ Oreocharis guangwushanensis

Taxon classificationPlantaePasseriformesParamythiidae

Z.L.Li & Xin Hong sp.nov.

902AD065-BF4E-50AD-A47D-93F1EB99DCEA

urn:lsid:ipni.org:names:77300515-1

Fig. 1 , Table 1

#### Diagnosis.

*Oreocharisguangwushanensis* morphologically resembles *O.ronganensis* and *O.reticuliflora*. The new species is vegetatively similar to *O.ronganensis*, but it differs from the latter in that the adaxial lip is 2-lobed to or above the middle (*vs.* lobed to near base), ovary with white pubescence (*vs.* glabrous), anthers in pairs (*vs.* anthers free), shorter tube (9–14 mm *vs.* 20 mm), shorter pistil (5 mm *vs.*12–15 mm) and filaments strongly twisted and bent at the top (*vs.* linear, straight). The new species resembles *O.reticuliflora* in habit, flower tube and the shape of the calyx, but differs by its pink limbs (*vs.* limbs with a network of violet stripes), filaments strongly twisted and bent at the top (*vs.* linear, straight), anthers in pairs (*vs.* anthers free), ovary with white pubescence (*vs.* glandular-pubescence) and shorter pistil (5 mm *vs.* 10–12 mm).

**Figure 1.**
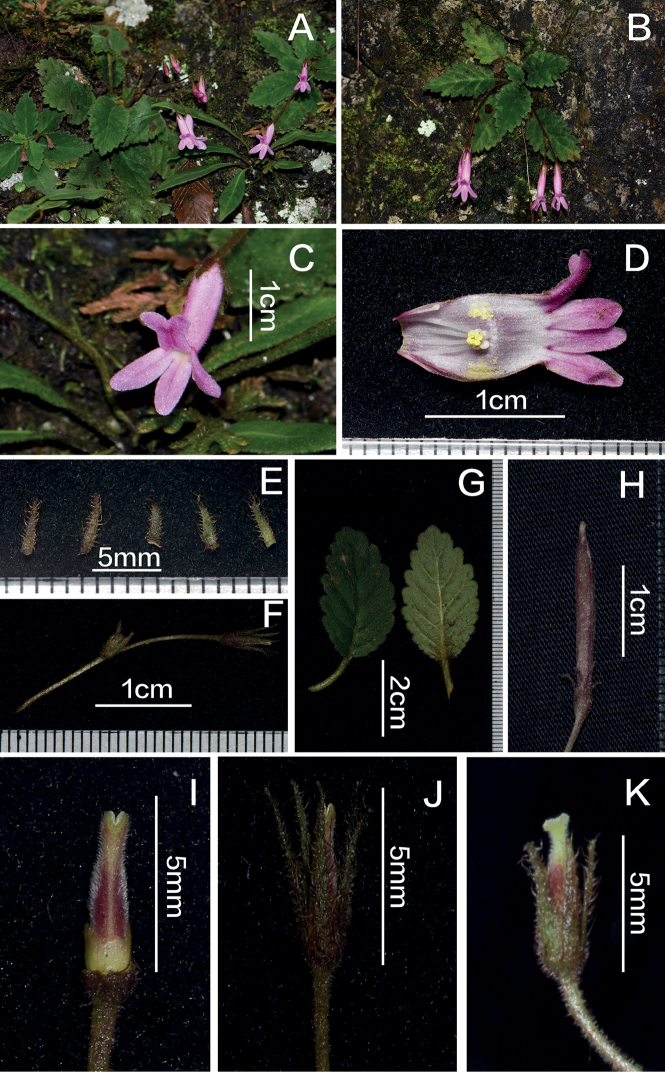
*Oreocharisguangwushanensis* Z.L.Li & Xin Hong **A** flowering plants in natural habitat **B** plant with pair-flowered cymes **C** corolla front view **D** opened corolla **E** dissected calyx lobes, outside brown with rusty strigose indumentum (3 left), inside green with brown pilose indumentum on margins (2 right) **F** peduncle, pedicel and young lateral branch **G** leaves (left: adaxial surface, right: abaxial surface) **H** capsule **I** immature pistil **J** immature pistil with calyx **K** mature pistil with calyx.

#### Type.

China. Sichuan province: Guangwushan Provincial Nature Reserve, 32°39'N, 106°45'E, 988 m a.s.l., growing on limestone cliffs near a river, 31 July 2020, flowering and fruiting, Hai Jun Ma, *MHJ 21073102* (holotype: IBK; isotype: AHU).

**Table 1.** Diagnostic character differences between *Oreocharisguangwushanensis* and its morphologically close relatives *O.ronganensis* and *O.reticuliflora*.

Character	*O.guangwushanensis* Z.L.Li & Xin Hong	*O.ronganensis* (K.Y.Pan) Mich.Möller & A.Weber	*O.reticuliflora* Li H. Yang & X.Z.
**Cymes**	1–3 (–6)-flowered	4–10-flowered	4–14 (–22)-flowered
**Tube**	9–14 mm long	ca. 20 mm long	12–15 cm long;
**Corolla**	corolla pink, with a single stripe in the middle of lower lobes	corolla pink, without stripes	corolla blue-purple, with a network of violet stripes on each lobe
**Adaxial lip 2-lobed**	adaxial lip 2-lobed to or above the middle	adaxial lip lobed to near base	adaxial lip lobed to near base
**Filament attachment**	2 mm from corolla base	8–10 mm from corolla base	6–8 mm from corolla base
**Stamens and anthers**	filaments strongly twisted and bent at the top, anthers in pairs	filaments linear, anthers free	filaments linear, anthers free
**Ovary**	white pubescent	glabrous	glandular-pubescent
**Pistil at maturity**	6–7 mm long	12–15 mm long	10–12 mm long

#### Description.

Perennial herb. ***Leaves*** basal, spirally arranged; petiole 2–10 cm long, densely brown pilose and pubescent; lamina thick herbaceous, oblong to lanceolate, 4–8 × 1.6–5 cm, base slightly asymmetrical, wedge-shaped to round, margin serrate, apex obtuse to acute, concave adaxially, dark green, densely brown pubescent; prominent abaxially, light green, densely pubescent, sparsely rust-brown long pilose; ca. 5–6 pairs of lateral veins on each side of the mid rib. ***Cymes*** 1–3, axillary, pair-flowered, 1–3 branches, 1–3 (–8) flowers; peduncles 4–10 cm long, densely rusty glandular-puberulent and sparsely strigillose; bracts 2, opposite, lanceolate, ca. 4 × 0.9 mm, densely brown pilose. Pedicel 1.5–5 cm long, densely rust-brown pilose and pubescent. Calyx 5-lobed to base, narrowly lanceolate, apex acute, ca. 5 × 0.9 mm, outside brown with brown strigose indumentum, inside green, brown pilose, most densely on the margins. ***Corolla***, pink, 1.8–2.6 cm long, densely glandular pubescent on both sides; tube cylindrical, constricted at the throat, slightly upwards curved, 9–14 mm long, 3 mm in diameter at the mouth; limb distinctly 2-lipped; adaxial lip 2-lobed to or above the middle, lobes 2 mm long, apex suborbicular, curving left and right, red stripe in the middle of the two fused upper lobes; abaxial lip 3-lobed to the base, lobes 6 mm long, oblong, with a red line in the middle of the lobes. ***Stamens*** 4, in pairs, glabrous, adnate to ca. 1–2 mm above the corolla base; filaments 5–7 mm long, strongly twisted and bent at the top, bent close to 270°, hooked, with white glands at the base, filaments of anterior pair 5–6 mm long, of posterior pair 6–7 mm long. Anthers yellow, ca. 0.8 mm long, fused by their entire adaxial surfaces. Staminode 1, linear, ca. 3 mm long, top slightly enlarged, adnate to ca. 2 mm above the corolla base; disc ring-like, about 1.5 mm high, with repand margin. ***Pistil*** mature ca. 6–7 mm long, at flower opening immature ca. 4.5 mm long, ovary linear-oblong, 2 mm long, reddish-brown, densely white pubescent. Style at pistil maturity ca. 1 mm long, densely pilose. Mature stigma with 2 lobes ca. 0.6 mm long, light green; ***Capsule*** brownish-red, ca. 2–2.5 cm × 0.2–0.3 cm, sparsely pubescent, narrowly oblong.

#### Phenology.

Flowering from May to July, fruiting from June to August.

#### Etymology.

The specific epithet is derived from the type locality, Guangwushan Provincial Nature Reserve, Sichuan province, China.

#### Vernacular name.

Guāng Wù Shān Mǎ Líng Jù Tái (Chinese pronunciation); 光雾山马铃苣苔 (Chinese name).

#### Distribution and habitat.

The new species has so far been found only in the type locality, Guangwushan Provincial Nature Reserve, Sichuan province, China. In the nature reserve, the average temperature is 16.2 °C, while the average annual precipitation has been calculated as ca. 1200 mm. The forest where *O.guangwushanensis* occurs is a monsoon evergreen broad-leaved forest.

#### Preliminary conservation assessment.

Currently, the new species has been observed only from the type locality. After two years of careful investigation, only two small populations of *O.guangwushanensis* have been found. They are less than 200 m apart. In total, ca. 200 mature individuals were present within 4 km^2^ (AOO). One population of no more than 40 individuals was growing at a higher altitude of 1124 m on a boulder near a road; the other population on a limestone wall at lower altitude 988 m by a river, close to the scenic area of the Guangwushan Provincial Nature Reserve which attracts many visitors. The locations of both populations are easily accessible and frequently passed by tourists, and thus, the number of individuals is likely to be detrimentally affected. The natural habitat could be disturbed or changed by human activities such as road expansion and other building construction. Following the IUCN Red List Categories and Criteria (IUCN 2019), the new species is provisionally assessed as Critically Endangered [CR B2ab(iii)].

## Supplementary Material

XML Treatment for Oreocharis guangwushanensis
